# Expansion and Diversification of MFS Transporters in *Kluyveromyces marxianus*

**DOI:** 10.3389/fmicb.2018.03330

**Published:** 2019-01-10

**Authors:** Javier A. Varela, Martina Puricelli, Noemi Montini, John P. Morrissey

**Affiliations:** School of Microbiology, Centre for Synthetic Biology and Biotechnology, Environmental Research Institute, APC Microbiome Institute, University College Cork, Cork, Ireland

**Keywords:** sugar transport, genome evolution, gene duplication, *HGT1*, hexose transport, *RAG1*, *KHT1*, major facilitator superfamily

## Abstract

In yeasts, proteins of the Major Superfamily Transporter selectively bind and allow the uptake of sugars to permit growth on varied substrates. The genome of brewer’s yeast, *Saccharomyces cerevisiae*, encodes multiple hexose transporters (Hxt) to transport glucose and other MFS proteins for maltose, galactose, and other monomers. For sugar uptake, the dairy yeast, *Kluyveromyces lactis*, uses Rag1p for glucose, Hgt1 for glucose and galactose, and Lac12 for lactose. In the related industrial species *Kluyveromyces marxianus*, there are four genes encoding Lac12-like proteins but only one of them, Lac12, can transport lactose. In this study, which initiated with efforts to investigate possible functions encoded by the additional *LAC12* genes in *K. marxianus*, a genome-wide survey of putative MFS sugar transporters was performed. Unexpectedly, it was found that the *KHT* and the *HGT* genes are present as tandem arrays of five to six copies, with the precise number varying between isolates. Heterologous expression of individual genes in *S. cerevisiae* and mutagenesis of single and multiple genes in *K. marxianus* was performed to establish possible substrates for these transporters. The focus was on the sugar galactose since it was already reported in *K. lactis* that this hexose was a substrate for both Lac12 and Hgt1. It emerged that three of the four copies of Lac12, four Hgt-like proteins and one Kht-like protein have some capacity to transport galactose when expressed in *S. cerevisiae* and inactivation of all eight genes was required to completely abolish galactose uptake in *K. marxianus*. Analysis of the amino acid sequence of all known yeast galactose transporters failed to identify common residues that explain the selectivity for galactose. Instead, the capacity to transport galactose has arisen three different times in *K. marxianus* via polymorphisms in proteins that are probably ancestral glucose transporters. Although, this is analogous to *S. cerevisiae*, in which Gal2 is related to glucose transporters, there are not conserved amino acid changes, either with Gal2, or among the *K. marxianus* galactose transporters. The data highlight how gene duplication and functional diversification has provided *K. marxianus* with versatile capacity to utilise sugars for growth.

## Introduction

*Kluyveromyces marxianus* is a yeast traditionally found in fermented dairy products such as cheese and kefir (Gethins et al., 2016; Coloretti et al., 2017; Tittarelli et al., 2018). Because of its connection with these products, *K. marxianus* has been granted QPS (Qualified Presumption of Safety) and GRAS (Generally Regarded as Safe) status in the EU and US, respectively, designating this yeast as safe to use in food applications (Fonseca et al., 2008). *K. marxianus* is also found during the production of other non-dairy products such as chocolate and tequila/mezcal (Lappe-Oliveras et al., 2008; Ho et al., 2014). In order to establish and often colonise these diverse environments, the yeast possesses the capacity to access a range of sugar polymers. This involves the production of specific enzymes that degrade complex substrates and transporters that mediate the uptake of monomers into the cell. For example, Cocoa bean fermentation, an essential step in chocolate production, is generally carried out by a consortium of indigenous species that includes *K. marxianus* (De Vuyst and Weckx, 2016). In this environment the pectinase enzyme produced by *K. marxianus* degrades the pectin present in the cocoa pulp allowing the establishment of other yeast species required in the fermentation process (Schwan and Wheals, 2004). *K. marxianus* is also present as part of the natural flora of the *Agave* plant, used to produce the alcoholic beverages tequila and mezcal (Escalante-Minakata et al., 2008). Degradation of complex fructans to simple sugars by the enzyme inulinase is essential in the establishment of *K. marxianus* in this niche. Besides utilising various substrates and producing relevant enzymes, *K. marxianus* also has other interesting physiological traits, uncommon to most yeasts (Lane and Morrissey, 2010). For example, it is able to grow at high temperatures (up to 50°C), exhibits the fastest growth rate among eukaryotes and naturally produces significant amounts of flavour molecules such as esters and fusel alcohols (Morrissey et al., 2015). This species is also used for some commercial applications, for example, the production of bioethanol from cheese whey and production of the flavour molecule 2-phenylethanol (Fonseca et al., 2008; Morrissey et al., 2015; Varela et al., 2017a).

The prospect of further developing *K. marxianus* for cell-factory applications has motivated researchers to sequence the genome of several strains (Jeong et al., 2012; Suzuki et al., 2014; Inokuma et al., 2015; Lertwattanasakul et al., 2015; Ortiz-Merino et al., 2018). A comparison of these genomes showed that *K. marxianus* displays a high level of genome variation at a structural and sequence level. Divergence in DNA sequence was found to be relativity high with up to 3% divergence between some isolates. Also, different strains were found to exist in haploid, diploid, and triploid states (Ortiz-Merino et al., 2018). This high degree of variation between genomes is not surprising considering previous observations of phenotypic diversity across isolates (Lane et al., 2011). Lactose utilisation, for example, is a variable trait with some strains displaying good and poor growth on lactose (Carvalho-Silva and Spencer-Martins, 1990; Lane et al., 2011). This phenomenon is due to polymorphisms in the *LAC12* gene, which encodes a lactose permease that mediates lactose uptake into the cell. Strains that display good and poor lactose consumption carry functional (*LAC12*-B) and non-functional *(LAC12*-A) alleles of the lactose transporter, respectively (Varela et al., 2017b). The sequence differences between the two alleles translate into 11 amino acid changes in the protein sequence that are sufficient to alter the function of the transporter (Ortiz-Merino et al., 2018). Unbiased analyses of genome sequences showed that isolates from dairy environments are diploid or triploid and carry the *LAC12-*B variant while non-dairy strains are haploid and contain the *LAC12*-A haplotype that encodes the non-functional lactose transporter (Ortiz-Merino et al., 2018). Although this analysis was performed in a relatively small number of genomes it raises interesting questions regarding the evolution of lactose utilisation in *K. marxianus*.

Information on the transport of other sugars, such as glucose and galactose, is limited in *K. marxianus*. Early experiments established the existence of three carbon-repressible proton-sugar transporters that mediate glucose/galactose, fructose, and lactose uptake (De Bruijne et al., 1988). Induction of the different systems was shown to depend on the carbon source used to grow the cells: in yeast cells grown on lactose, the activity of a high-affinity proton-glucose transporter was detected whereas a low-affinity glucose transporter was present in cells grown in glucose. Galactose was only taken up by cells grown in lactose and not by glucose-grown cells, which indicates that the galactose transport system requires induction by lactose or presumably galactose (Gasnier, 1987). The genes encoding these transporters were not identified in *K. marxianus* but both low and high-affinity glucose uptake systems were described the related yeast, *Kluyveromyces lactis* (Wésolowski-Louvel and Goffrini, 1992; Billard et al., 1996; Schaffrath and Breunig, 2000). In most strains, the low-affinity system is encoded by two tandemly-arrayed genes, *KHT1* and *KHT2* but some strains carry a single gene, *RAG1*, that arose from a recombination event between *KHT1* and *KHT2* (Weirich et al., 1997). The high affinity transport system is encoded by *HGT1*, which was found to exist as a single copy (Billard et al., 1996). Expression of this gene was found to be constitutive whereas expression of *KHT1* and *KHT2* genes was glucose inducible (Milkowski et al., 2001). Interestingly, a strain carrying null mutations for both of the uptake systems is still able to grow on glucose, which points out to the existence of another glucose transporter not yet identified (Milkowski et al., 2001). Galactose uptake was also investigated in *K. lactis* where the *LAC12* gene was found to play a role as a low-affinity galactose transporter (Riley et al., 1987). In that study, strains mutated in *LAC12* were greatly impaired in galactose uptake, which led to the idea that galactose was exclusively transported via Lac12p. Conversely, further studies on the topic showed that the high-affinity glucose carrier, *HGT1*, also acts as a high-affinity galactose transporter in *K. lactis* (Baruffini et al., 2006). Disruption of both *LAC12* and *HGT1* abolished galactose growth completely, ruling out the existence of other galactose transporters. It was later concluded that *LAC12* and *HGT1* encode a low-affinity and high-affinity galactose system, respectively (Baruffini et al., 2006).

Previously, we showed that there are four copies of the *LAC12* gene *K. marxianus* but only one of these encodes a functional lactose transporter (Varela et al., 2017b). All the copies of *LAC12* are found in sub-telomeric regions and it was not determined whether the additional copies could function in the transport of sugars other than lactose, The recent implementation of the CRISPR-Cas9 system in *K. marxianus* creates new opportunities to study the molecular basis of sugar uptake in this yeast (Löbs et al., 2017; Nambu-Nishida et al., 2017; Juergens et al., 2018). In this study, we used comparative genomics to identify putative sugar transporters in multiple *K. marxianus* strains. This revealed that there was also expansion in the number of genes that were homologous to the *K. lactis KHT1/2* and *HGT1* genes. Functional analysis to establish the capacity of these duplicated genes to transport galactose revealed a high degree of functional overlap. This work highlights the existence of a species-specific gene expansion and raises fascinating questions about the evolution of galactose and lactose utilisation in *K. marxianus*.

## Materials and Methods

### Strains and Growth Conditions

The yeast strains used in this study are listed in Table 1. *K. marxianus* CBS6556 and NBRC1777 were obtained from the Westerdijk Fungal Biodiversity Institute and the NITE Biological Research Centre culture collections, respectively. *Saccharomyces cerevisiae* EBY.VW4000 was kindly provided by Dr. Eckhard Boles, Goethe University Frankfurt, Germany. *K. marxianus* strains were typically grown in YPD medium (10 g L^-1^ yeast extract, 20 g L^-1^ peptone, 20 g L^-1^ glucose). When used in transformation experiments, NBRC1777 was plated onto YPD plates containing 200 μg mL^-1^ of Hygromycin B (Sigma-Aldrich, St. Louis, MI, United States). *K. marxianus* mutants were grown on mineral media (MM) supplemented with 20 and 0.1 g L^-1^ glucose, or galactose (Fonseca et al., 2007). *S. cerevisiae* EBY.VW4000, used for heterologous expression of sugar transporters (Wieczorke et al., 1999), was maintained in synthetic complete (SC) medium (1.7 g L^-1^ yeast nitrogen base, 5 g L^-1^ ammonium plus synthetic complete drop-out lacking uracil and or L-leucine) (Formedium, Norfolk, United Kingdom) supplemented with 20 g L^-1^ maltose. For functional analysis the EBY.VW4000 strains expressing the different transporters were grown on SC – maltose, washed, serially diluted and spotted onto SC plates containing glucose or galactose to a final concentration of 20 or 1 g L^-1^, as described in the text. Yeast strains were grown at 30°C with 200 rpm agitation unless otherwise mentioned. *E. coli* DH5α was used for cloning purposes. The strain was maintained in LB medium (5 g L^-1^ yeast extract, 10 g L^-1^ bactopeptone, 10 g L^-1^ NaCl) supplemented with 100 μg mL^-1^ ampicillin when required.

**Table 1:** Yeast strains used in this study.

Strain	Genotype	Mutation coordinates
*S. cerevisiae* EBY.VW4000	EBY.VW1000:: stl1v::loxP agt1v::loxP ydl247wv::loxP yjr160cv::loxP	–
*K. marxianus* CBS6556	Wild-type strain	–
*K. marxianus* NBRC1777	Wild-type strain	–
NBRC1777 Δ*lac*	*Kmlac12 lac12-2 lac12-4*	*KmLAC12:*CHR III 16,869.-T *LAC12-2:* CHR III 1,557,231.-T
		*LAC12-4:* CHR VIII 12,834.-GT
NBRC1777 Δ*E03670*	ΔE03670	E03670: CHR V 782,125.-T
NBRC1777 Δ*hgt75*	Δ*hgt*	CHR I Δ1,131,464-1,145,439
NBRC1777 Δ*lac Δ*E03670	*Kmlac12 lac12-2 lac12-4* E03670	*LAC12* genes: same as Δ*lac*
		E03670: CHR V 782,125.-T
NBRC1777 Δ*lac*Δ*hgt*	*Kmlac12 lac12-2 lac12-4*Δ*hgt*	*LAC12* genes: same as Δ*lac* *HGT:* CHR I Δ1,131,464-1,145,439
NBRC1777 Δ*hgt*ΔE03670	Δ*hgt*ΔE03670	*HGT:* CHR I Δ1,131,464-1,145,439 E03670: CHR V 782,125.-T
NBRC1777 Δ*lac*Δ*hgt*ΔE03670	*Kmlac12 lac12-2 lac12-4*Δ*hgt*ΔE03670	*LAC12* genes: same as Δ*lac* *HGT:* CHR I Δ1,131,464-1,145,439
		E03670: CHR V 782,125.-T


*The genotype of the *K. marxianus* mutants constructed in this study is shown. Mutation coordinates refer to the position where the mutations are located in the NBRC1777 genome. Single or double nucleotide deletions are represented by a (-) symbol followed by the nucleotide/s deleted. All mutations result in frameshifts that produce early stop codons.*

### Identification of Potential Sugar Transporters

A genome-wide identification of sugar transporters was performed in the *K. marxianus* CBS6556 and *K. lactis* CBS2359 genomes (Accession Nos. PRJNA89605 and PRJNA12377, respectively). The predicted proteomes from the two species were independently compared against the TransportDB 2.0 database to identify possible candidates (Elbourne et al., 2017). After filtering out hits with low identity (<35%) and coverage (<80%), the resulting sequences were submitted to the TMHMM server^1^ for the prediction of transmembrane domains. All sequences containing two or more transmembrane domains were considered as potential transporters. In order to classify these transporters, their sequences were aligned against the PFAM database (Finn et al., 2014) using HMMER v 3.0 (Finn et al., 2011). Sequences belonging to the MFS superfamily were extracted, aligned using MUSCLE 3.8 (Edgar, 2004) and then used to construct a maximum-likelihood tree using PhyML 3.1 (Guindon et al., 2010). Tree visualisation was carried out using the ETE 3 toolkit (Huerta-Cepas et al., 2016).

To compare the structure of the *KHT* and *HGT* loci across species, the publicly available *Kluyveromyces* genomes were aligned and visualised using progressiveMauve (Darling et al., 2010). *K. marxianus* genomes from different strains (CBS6556, NBRC1777, UFV-3, CBS397, CBS4857, and DMKU3) were also analysed using this software.

### Heterologous Expression of Sugar Transporters

Plasmids and primers used in this study are listed in Tables 2, 3, respectively. The plasmid p426 was used to clone and express the *KHT* and *HGT* genes in *S. cerevisiae*. The p426 backbone, *ScTEF1* promoter, *ScCYC1* terminator and the KMXK_E03650 gene from *K. marxianus* CBS6556 were amplified by PCR with primers containing overlapping regions. PCR products were then purified and assembled using a Gibson assembly kit (New England Biolabs, Inc., Ipswich, MA, United States). The resulting plasmid, which expresses the KMXK_E03650 gene under the control of the *ScTEF1* promoter, was used as a backbone for cloning the rest of the transporters. The primers p426-TEF-F and p426-CYC-R, were used to PCR amplify the p426- KMXK_E03650 plasmid from the *ScTEF1* promoter to the *ScCYC1* terminator (excluding the KMXK_E03650 gene). Then, purified PCR products from the rest of the CBS6556 transporters were individually combined with the purified p426 backbone and cloned by Gibson assembly. The *KHT* genes KMAR_50343 and KMAR_50344, absent in CBS6556, were PCR amplified from NBRC1777 and cloned following the same procedure. All the PCR reactions were performed using DNA Phusion Polymerase (New England Biolabs, Inc., Ipswich, MA, United States). Plasmids were finally introduced into *S. cerevisiae* EBY.VW4000 by transformation using the LiAc/SS carrier DNA/PEG method (Gietz and Schiestl, 2007). Expression of the *LAC12* genes was also tested using plasmids previously described (Varela et al., 2017b). After transformations, the cells carrying the p426-*KHT/HGT* and *LAC12* plasmids were plated onto SC media lacking uracil and leucine, respectively.

**Table 2:** Plasmids used in this study.

Plasmid	Source
p426	Addgene #43803
p426-TEF1-E03650	This study
p426-TEF1-E03660	This study
p426-TEF1-E03670	This study
p426-TEF1-E03680	This study
p426-TEF1-E03690	This study
p426-TEF1-50344	This study
p426-TEF1-50343	This study
p426-TEF1-A02920	This study
p426-TEF1-A02930	This study
p426-TEF1-A02940	This study
p426-TEF1-A02950	This study
p426-TEF1-A02960	This study
p426-TEF1-E00380	This study
pGREG-505 – TEF1	Varela et al., 2017b
pGREG-505 – TEF1 – *KmLAC12* – CBS 6556	Varela et al., 2017b
pGREG-505 – TEF1 – *KmLAC12* – CBS 397	Varela et al., 2017b
pGREG-505 – TEF1 – *LAC12-2* – CBS 397	Varela et al., 2017b
pGREG-505 – TEF1 – *LAC12-3* – CBS 397	Varela et al., 2017b
pUDP002	Juergens et al., 2018
pUCC001	This study
pUCC001-*KmLAC12*	This study
pUCC001-*LAC12-2*	This study
pUCC001-*LAC12-4*	This study
pUCC001-E03670	This study
pUCC001-*HGT*	This study


**Table 3:** Primers used in this study.

Primer	Sequence
**Heterologous expression of *KHT* and *HGT* genes**	
p426-F	TTCGCCAGCTGGCGTAATAG
p426-R	GGGAGAGGCGGTTTGCGTATTG
*ScTEF1*-F	gcccaatacgcaaaccgcctctccc CATAGCTTCAAAATGTTTCTACTC
*ScTEF1*-R	cagcttcggacatCTTAGATTAGAT TGCTATGCTTTC
*ScCYC1*-F	tatgatgaaataaTCATGTAATTA GTTATGTCACGC
*ScCYC1*-R	cttcgctattacgccagctggcgaa GCAAATTAAAGCCTTCGAGC
E03650-F	aatctaatctaagATGTCCG AAGCTGCTGGTTTAC
E03650-R	actaattacatgaTTATTTCATC ATAGCCTTGTACCATG
p426-TEF-F	CTTAGATTAGATTGCTATGCTTTC
p426-CYC-R	TCATGTAATTAGTTATGTCACGC
E03660-F	gcatagcaatctaatctaagATGTCTGA AGCTGCCGCTG
E03660-F	tgacataactaattacatgaTTAATGCT TCATCATGGCCTTG
E03670-F	gcatagcaatctaatctaagATGTCTGA AGAAGCTGCATTACAG
E03670-R	tgacataactaattacatgaTTAGGACG TCATGCGCTTG
E03680-F	gcatagcaatctaatctaagATGTCCA ATCAATTAACGG
E03680-R	tgacataactaattacatgaTTAGTTC TTCTTGAAGGACATG
E03690-F	gcatagcaatctaatctaagATGTCTA ATCAATTGACGGC
E03690-R	tgacataactaattacatgaTTATTTC AAAGAAATCCTCTTG
50343-F	gcatagcaatctaatctaagATGTCTGAA GCTGCTGCTGATTTACA
50343-R	tgacataactaattacatgATTAATGCTT CATCATGGCCTTGTACCA
50344-F	gcatagcaatctaatctaagATGTCTGAA GCTGCCGCTGA
50344-R	gcgtgacataactaattacatgaCTCGA GGTCGACTTAATGCTTCAACAA AGCCTTGTACCAT
A02920-F	gcatagcaatctaatctaagATGACTT TAAAAGATAAACTATTGCTCC
A02920-R	tgacataactaattacatgaCTAGAC CGAGCTGCTGCTATTAG
A02930-F	gcatagcaatctaatctaagATGTC ATTCTTAGACAAGAAAAC
A02930-R	tgacataactaattacatgaTCAAA CATTGTCATTCCTTAC
A02940-F	gcatagcaatctaatctaagATGTC ATTTAAAGACAAGTTTTC
A02940-R	tgacataactaattacatgaTTAAA CGCTGTTACCAGAG
A02950-F	gcatagcaatctaatctaagATGAA ACAATTCGCTACG
A02950-R	tgacataactaattacatgaTTATA CGCGAGAGTCGTTC
A02960-F	gcatagcaatctaatctaagATGTC ATTGAAAGACAAGATTTTG
A02960-R	tgacataactaattacatgaTTAGT TAGAGTTTGAGTTTGAGTTG
**Construction of pUCC001**	
pUDP002-F	tcggacgagcttactcgtttcgtc ctcacggactcatcagGTTTGTTTGT TTATGTGTGTTTATTC
pUDP002-R	gcgccggctgggcaacatgcttcg gcatggcgaatgggacCACAGGCCCC TTTTCCTTTG
HH-BSA-HDV-F	CTGATGAGTCCGTGAGGACGA AACGAGTAAGCTCGTCCGAGACCTG CGGAGGTCTCCGTTTTAGAGCTAGAA ATAGCAAGTTAAAATAAGGCTAGT CCGTTATCAACTTGAAAA AGTGGCACC
HH-BSA-HDV-R	GTCCCATTCGCCATGCCGAAG CATGTTGCCCAGCCGGCGCCAGCGA GGAGGCTGGGACCATGCCGGCCA AAAGCACCGACTCGGTGCCACT TTTTCAAGTTGATAACGGACTAGC CTTATTTTAACTTG
**Construction of CRISPR plasmids**	
*Km*LAC12-gRNA-F	CGTC**TCGTCTCTTGGATTTCTTCA**
*Km*LAC12-gRNA-R	AAAC**TGAAGAAATCCAAGAGACGA**
*LAC12-2*-gRNA-F	CGTC**CGCTAAAGCCGCGCCTAGAG**
*LAC12-2*-gRNA-R	AAAC**CTCTAGGCGCGGCTTTAGCG**
*LAC12-4*-gRNA-F	CGTC**GTGCTGCATTAGCATTATCA**
*LAC12-4*-gRNA-R	AAAC**TGATAATGCTAATGCAGCAC**
E03670*-*gRNA-F	CGTC**GTACGTCTTGGTACCGTAGT**
E03670*-*gRNA-R	AAAC**ACTACGGTACCAAGACGTAC**
*HGT-*gRNA-F	CGTC**CGCTCCAACCTGGGGTAT**
*HGT-*gRNA-R	AAAC**ATACCCCAGGTTGGAGCG**
BSA-R	TACACGCGTTTGTACAGAAAAAAAA GAAAAATTTGA
**Diagnostic primers to check for CRISPR-*Cas9*-induced mutagenesis**	
Diag*KmLAC12*-F	TTTGGTTGGTTAATCCCAGA ATCTCCAAG
Diag*KmLAC12*-R	GGATGCCTTTCTTGGGTTTGGAG
Diag*LAC12-2*-F	CGGGTCTCGTCTGTATTTTCGGT
Diag*LAC12-2*-R	TAGGCTGCATAGGAGTAA ATGCAAACG
Diag*LAC12-4*-F	TTGTCGTCTCATCTGTGGTCACG
Diag*LAC12-4*-R	TTCATAGCTCTGGGTGCGGC
DiagE03670-F	GAAGTTGACAATACCCAAG ACGATGGA
DiagE0367*0*-R	CTCTGGTTTGGGTGTTGGTGGT
Diag*HGT*-F	CTCATCTTGGATAAAATTTGCTTG
Diag*HGT*-R	ATTAAAAGGGAGAGAGGGTGG


*The section of the primer that anneals to the target sequence is shown in uppercase. Sequences in lower case are homologous to the plasmid where the sequences were cloned. Sequences in bold correspond to the protospacer sequences for CRISPR-Cas9 experiments.*

### Construction of *K. marxianus* Mutants

The CRISPR-Cas9 system was used to disrupt the genes encoding galactose transporters in *K. marxianus* NBRC1777, including the *LAC12, HGT* genes and the *KHT* gene, KMXK_E03670. The pUDP002 plasmid, containing the *Cas9* gene and the elements required to express the guide RNA (gRNA) molecule in *K. marxianus* (Juergens et al., 2018), was modified to simplify cloning of target sequences. Long overlapping oligonucleotides encoding the hammerhead ribozyme, a BsaI cloning site, the gRNA structural part and the HDV ribozyme were chemically synthesised (Integrated DNA Technologies, Coralville, IA, United States) and annealed in a thermocycler machine. In parallel, the pUDP002 plasmid was amplified by PCR using the pUDP002-F and pUDP002-R primers. The 205 bp DNA-duplex and the plasmid backbone were assembled via Gibson assembly to yield the pUCC001 plasmid (Supplementary Figure S1). Design of the gRNA sequences targeting the genes of interest was carried out using the software sgRNAcas9 (Xie et al., 2014). Complementary oligonucleotides containing the gRNA sequences and specific overhangs (5′-CGTC-3′ and 5′-AAAC-3′) were annealed, phosphorylated and cloned into pUCC001 by Golden Gate assembly, as described previously (Vyas et al., 2015; Ng and Dean, 2017). Presence of the target sequences in the pUCC001 plasmid was confirmed by colony PCR using the forward target primer and BSA-R. Plasmids were introduced into NBRC1777 by transformation using the LiAc/SS carrier DNA/PEG method and transformants checked by PCR and DNA sequencing. In order to eliminate the pUCC001 plasmid from the mutants, the strains were grown on YPD broth overnight without selection and diluted 1/10 into fresh YPD next morning. The procedure was repeated twice and then the strains were grown on YPD medium supplemented with Hygromycin B to confirm plasmid loss. To construct strains carrying mutations at additional loci, the procedure was repeated sequentially with one gene being mutated at a time. Primers used for cloning the gRNA molecules and checking transformants are listed in Table 3. The genotype of all the *K. marxianus* mutants constructed in this study is shown in Table 1.

## Results

### Extra *LAC12* Copies Encode Galactose Transporters

**FIGURE 1:**
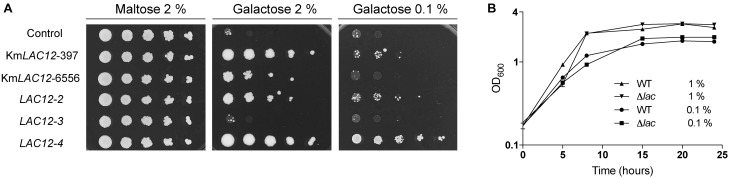
Role of the *LAC12* genes in galactose transport. **(A)** Heterologous expression of the *Kluyveromyces marxianus LAC12* genes in *Saccharomyces cerevisiae* EBY.VW4000. Yeast strains were grown on SC maltose to an OD_600_ of 2 then washed, diluted serially and spotted onto SC plates supplemented with maltose, galactose or glucose, as indicated. Plates were incubated for 5 days at 30°C. The *LAC12-2* and *LAC12-4* genes were obtained from *K. marxianus* CBS397, as described by Varela et al. (2017b). **(B)** Growth phenotype of *K. marxianus*Δ*lac* mutant carrying null mutations for the *KmLAC12, LAC12-2*, and *LAC12-4* genes. NBRC1777 and the Δ*lac* strain were grown overnight on MM supplemented with 1 or 0.1% galactose and then transferred to fresh medium containing the same amount of sugar to an OD_600_ of 0.1. Growth was recorded every 6 h using a spectrophotometer. The experiment was conducted in triplicates.

It was recently shown that *K. marxianus* contains four copies of the lactose permease gene with only *KmLAC12* encoding a functional lactose transporter (Varela et al., 2017b). Lactose transport by Lac12p appears to be a trait that evolved in a particular dairy lineage of *K. marxianus* (Ortiz-Merino et al., 2018) and it was hypothesised that the proteins encoded by the *LAC12* genes might have other substrates. Because the *K. lactis* Lac12p protein is known to transport lactose and galactose (Riley et al., 1987), it was decided to test whether the additional *K. marxianus* Lac12 proteins were also galactose transporters. To test this, the different *LAC12* genes were expressed in *S. cerevisiae* EBY.VW4000 (Figure 1A), a yeast strain that has been subjected to systematic disruption of all known hexose transporters (Wieczorke et al., 1999). As previous work demonstrated that Lac12p from the Lac^+^ and Lac^-^ lineages of *K. marxianus* were functionally different, both *KmLAC12_397_* (lac^+^) and *KmLAC12_6556_* (lac^-^) were tested. The *S. cerevisiae* strains expressing *KmLAC12, LAC12-*2, and *LAC12-*4 grew in 2% galactose medium indicating that these proteins could transport galactose into the cell, though it was apparent that the CBS397 version of *LAC12* promoted better growth that the equivalent gene from CBS6556. To check whether the Lac12 proteins also supported growth where high-affinity galactose transport was required, the different strains were also grown on 0.1% galactose. In this case, only the strain expressing *LAC12*-4 exhibited strong growth. The *LAC12*-3 strain did not confer growth under any condition. Since these results suggested that *KmLAC12, LAC12*-2, and *LAC12*-4 encode galactose transporters, we then tested whether inactivation of all three genes in *K. marxianus* would generate a strain unable to grow using galactose as a sole carbon source. This entailed the sequential construction of single, double and triple mutants using a modified version of the previously published CRISPR-Cas9 system (Juergens et al., 2018). *K. marxianus* strain NBRC 1777 was used as this is a haploid strain in which CRISPR-Cas9 works efficiently. The three *LAC12s* were targeted using this system and indel mutations, inactivating the genes by producing premature stop codons, were obtained in all cases. The triple *lac12 lac12-2 lac12-4* mutant (designated *K. marxianus* NBRC1777 Δ*lac*), and the wild-type strain were grown in 1 and 0.1% galactose media but no major differences in growth between wild-type and mutants were observed on plates (data not shown) or in liquid (Figure 1B). This indicates that there is an additional galactose transporter(s) in *K. marxianus*, an idea compatible with previous studies in *K. lactis* where *HGT1* was reported to encode a high-affinity galactose transporter (Baruffini et al., 2006).

### The *KHT* and *HGT* Gene Family Is Expanded in *K. marxianus*

**FIGURE 2:**
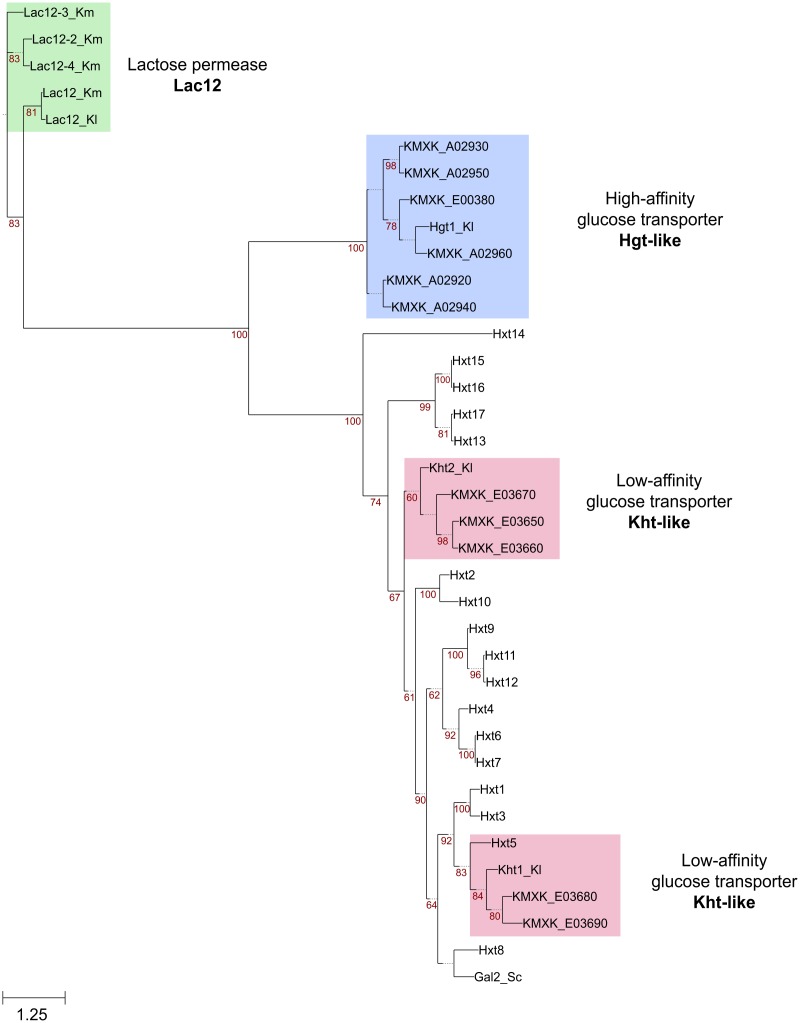
Phylogenetic tree of *K. marxianus* sugar transporters. The *K. marxianus* Kht, Hgt, and Lac12 protein sequences were aligned using MUSCLE and then used to construct a maximum-likelihood tree using PhyML with a bootstrapping value set to 500. The *Kluyveromyces lactis* Kht and Hgt sequences, and the Hxt sequences from *S. cerevisiae* were also used in this analysis. Branches with bootstrapping values over 60 are shown. The Kht and Hgt sequences are highlighted in red and blue boxes, respectively. Lac12 sequences are shown in green.

A genome-wide annotation of membrane transporters was performed to identify transporters of the major facilitator superfamily (MFS) that could potentially encode galactose transporters in *K. marxianus* CBS6556. The predicted proteomes of *K. marxianus* and *K. lactis* were compared against the TransportDB database, filtered by number of transmembrane domains, and categorised into protein families (Supplementary Table S1). Through this process, a total of 49 and 41 MFS transporters were identified in the *K. marxianus* and *K. lactis* proteomes, respectively. Interestingly, this analysis revealed that, compared to *K. lactis*, there has been an expansion of transporters of this superfamily in *K. marxianus* with five copies of the putative low-affinity glucose transporter Kht and six copies of the putative high-affinity glucose transporter Hgt1 identified. To gain a better understanding of the relationship between these sequences, the proteins were aligned, and a maximum-likelihood phylogenetic tree was constructed (Figure 2). The Hxt hexose transporter-like proteins (Hxt proteins) in *S. cerevisiae*, were also included in this analysis. Three of the Kht proteins (KMXK_E03650, KMXK_E03660, and KMXK_E03670) are related to the Kht2 transporter from *K. lactis*. In contrast, KMXK_E03680 and KMXK_E03690 are more similar to the *K. lactis* Kht1 protein and are related in sequence to the *S. cerevisiae* hexose transporter Hxt5. All the Hgt1-like transporters resemble to the *K. lactis* Hgt1p transporter with KMXK_A02960 being the closest in sequence to the *K. lactis* protein.

**FIGURE 3:**
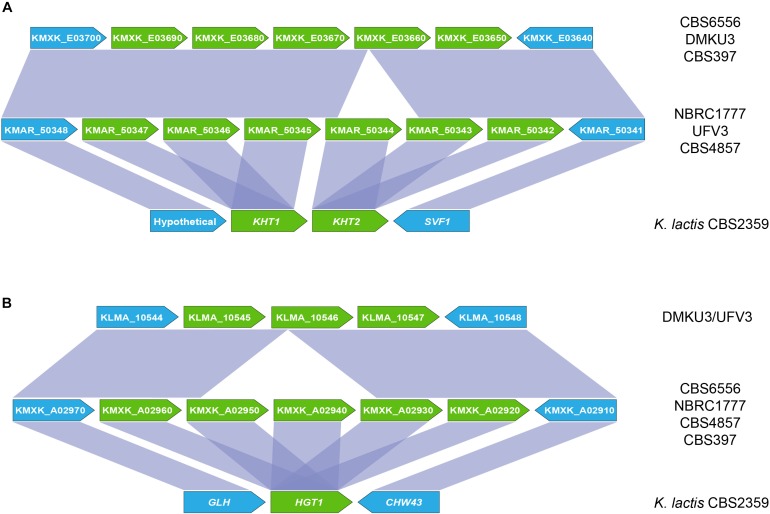
Organisation of the *KHT* and *HGT* genes in *K. marxianus* genomes. **(A)** Genomic context of the *KHT* genes in *K. marxianus* strains NBRC1777, UFV3 and CBS4857 (top), CBS6556, DMKU3 and CBS397 (middle) and rest of *Kluyveromyces* genomes, represented by *K. lactis* (bottom). **(B)** Position of the *HGT* genes in the CBS6556, NBRC1777, CBS397 and CBS4857 genomes (top), DMKU3 and UFV3 (middle), and *K. lactis* (bottom). Sequence conservation across the different regions is represented by purple areas connecting the genes in different genomes. Sugar transporter genes are shown in green. Genes flaking these clusters are shown in blue.

An inspection of the *K. marxianus* CBS6556 genome revealed that most of the additional *KHT* and *HGT* genes are present as tandem repeats, the sole exception being the *HGT1* homolog KMXK_E00380. To determine whether this expansion was unique to strain CBS6556, the *KHT* and *HGT* loci were compared across a set of publicly available *K. marxianus* genomes and other *Kluyveromyces* genomes (*K. lactis, K. aestuarii, K. wickerhamii*, and *K. dobzhanskii*) (Figure 3). In all species except *K*. *marxianus*, two *KHT* genes and a unique *HGT* gene were present. The expansion observed in strain CBS6556 was seen in all five additional *K. marxianus* strains assessed but the copy number was found to vary. While *K. marxianus* strains CBS6556, DMKU3, and CBS397 carry five copies of the *KHT* genes, strains NBRC1777, UFV3 and CBS4857 encode six copies of the transporter (Figure 3A). For consistency, the *K. marxianus* CBS6556 gene nomenclature is used throughout this paper, with Supplementary Table S2 listing the names for the orthologous genes in each of the other well-annotated *K. marxianus* genomes. An alignment of the NBRC1777 and CBS6556 sequences showed that the 5′ and 3′ sections of the KMXK_E03660 sequence are identical to KMAR_50344 and KMAR_50345, respectively, suggesting that KMXK_E03660 arose by a recombination event between two *KHT* genes (Supplementary Figure S2). A similar scenario was observed at the *HGT1* locus where some strains (NBRC1777, CBS6556, CBS397, and CBS4857) carry five and others (DMKU3 and UFV3) three copies of the gene (Figure 3B). A more detailed phylogenetic tree that includes all Hgt1 sequences shows a topology that is consistent with the presence of a single *HGT1* gene in ancestral *Kluyveromyces* species. This gene duplicated twice in *K. marxianus*, once to give rise to KMXK 0E00380 and once to give rise to the ancestor of KMXK 0A02920, KMXK 0A02930 KMXK 0A02940, and KMXK 0A02950 (data not shown). A comparison of the NBRC1777 and DMKU3 sequences established that a reduction in copy number occurred as a result of recombination between the second and fourth *HGT* genes, leading to a loss of the third gene (Supplementary Figure S3).

### Functional Analysis of the *KHT* and *HGT* Genes

**FIGURE 4:**
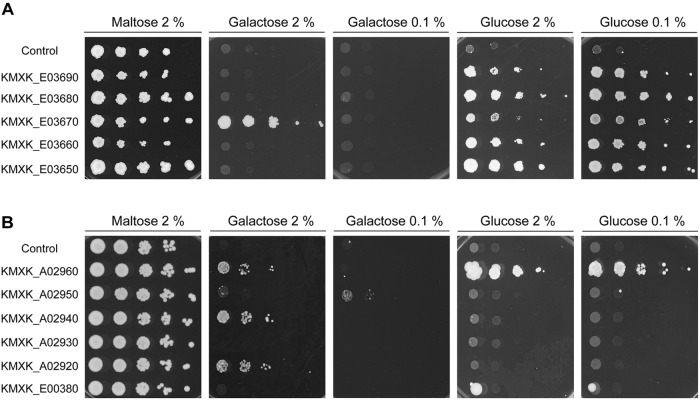
Functional analysis of the *KHT* and *HGT* genes in *S. cerevisiae* EBY.VW4000. *S. cerevisiae* strains expressing the *KHT* and *HGT* genes from CBS6556 were grown as previously described in Figure 1 and spotted onto SC plates containing maltose, galactose, or glucose. **(A)** *KHT* genes. **(B)** *HGT* genes.

The *KHT1/2* and *HGT1* orthologs in *K. lactis* were previously shown to encode glucose transporters, with Hgt1 also able to transport galactose into the cell. To functionally characterise the *K. marxianus* gene families, the *K. marxianus* CBS6556 genes were cloned and expressed in *S. cerevisiae* EBY.VW4000. The *S. cerevisiae* strains carrying the individual *K. marxianus KHT* and *HGT* genes were tested for growth on plates with the sole carbon source either glucose or galactose. Two sugar concentrations, 2 and 0.1% were used, as the lower concentration can establish whether the transporter has high affinity for the substrate (Figure 4). All the *S. cerevisiae* strains expressing *KHT* genes grew on both concentrations of glucose, indicating that all five of these proteins are high-affinity glucose transporters (Figure 4A). The only growth that was observed on galactose plates was with the strain expressing KMXK_E03670 on 2% galactose, showing that KMXK_E03670 can also function as a low-affinity galactose transporter. The pattern observed in strains expressing the *HGT* genes was very different (Figure 4B). Only KMXK_A02960 encoded a glucose transporter, whereas three genes, KMXK_A02960, KMXK_A02940, and KMXK_A02920, encoded proteins that could function as low-affinity galactose transporters, and the strain expressing KMXK_A02950 showed weak growth on 0.1% galactose.

**FIGURE 5:**
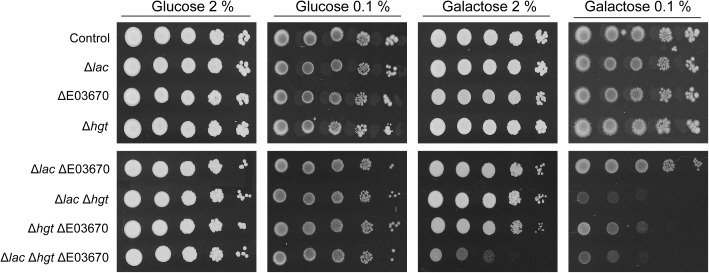
Phenotype of *K. marxianus* strains carrying mutations in galactose transport genes. The strains were grown on MM supplemented with 20 g/L to an OD_600_ of 2 then washed, diluted serially, and spotted onto MM plates supplemented with galactose or glucose, as indicated. The Δ*lac* strain contains mutations in the *KmLAC12, LAC12-*2 and *LAC12-*4 genes, the ΔE03670 strain contains a single nucleotide deletion in the E03670 gene and Δ*hgt* carries a deletion of the whole region containing the *HGT* genes. The precise genotype of these mutants is shown in Table 1. The wild-type strain NBRC1777 was used as a control in this experiment.

To definitively confirm that the *K. marxianus* genome encodes multiple galactose transporters, CRISPR-Cas9 was deployed to inactivate the genes encoding all of these potential transporters. The entire *HGT* locus was targeted for inactivation by using a single CRISPR gRNA that had target sequences in the first and last genes of the cluster and thus should lead to generation of double stranded breaks at each end. Repair of these breaks led to deletion of all functional *HGT1* copies at this locus and generated the strain that lacks any intact *HGT1*-like gene at this locus (Supplementary Figure S4). The strain retains KMXK_E00380, but this gene is not a galactose transporter (Figure 4). A CRISPR gRNA that successfully targeted the *KHT*-like gene, KMXK_E03670, was also tested and by sequential mutagenesis it was possible to make a set of *K. marxianus* mutants that lacked various combinations of the three functional *LAC12* genes (Δ*lac*), all five *HGT-*like genes (Δ*hgt*), and KMXK_E03670 (DE03670). These mutants were assessed for their ability to grow on medium where the sole carbon source was 2 or 0.1% glucose or galactose (Figure 5). None of the mutants was impaired in growth on glucose, which was not unexpected since all mutants still contain several of the Kht-like proteins, which were shown to transport glucose (Figure 4). On galactose, strains individually carrying the Δ*lac*, ΔE03670, or Δ*hgt* mutations did not show growth differences when compared to the wild-type strain, but some combinations of these mutations showed clear growth impairment. On 2% galactose, only growth of the strain inactivated in all putative galactose transporter genes (Δ*lac*ΔE03670 Δ*hgt*) was strongly impaired, corroborating the proposal that there is functional redundancy for galactose transport. On 0.1% galactose, all the strains carrying combinations of mutants were growth impaired to some extent, but the strongest phenotypes were in the triple mutant Δ*lac*ΔE03670 Δ*hgt* and the double mutant Δ*lac*Δ*hgt*, where growth was essentially abolished. A minor and intermediate growth impairment was seen in the Δ*lac*ΔE03670 and Δ*hgt*ΔE03670 strains, respectively.

**FIGURE 6:**
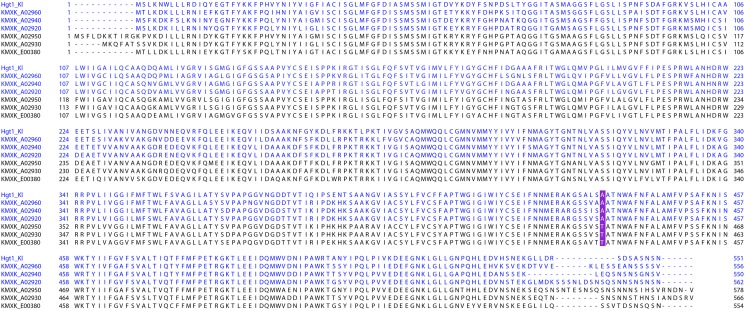
Multiple sequence alignment of the Hgt proteins. The Hgt sequences were aligned using MUSCLE 3.8. The Hgt1 sequence from *K. lactis* was also included in this comparison. The position where galactose and non-galactose transporters differ is marked in purple in the alignment (position 435 in KMXK_A02960). This residue is located in the twelfth transmembrane domain, according to TMHMM predictions. Sequences encoding galactose transporters are shown in blue.

The Lac12, Hgt, and Kht protein sequences were compared to identify amino acid residues potentially involved in galactose transport. Previous studies of the Gal2 transporter from *S. cerevisiae* previously identified three aromatic amino acid residues that are critical for galactose recognition and transport, Tyr^352^, Tyr^446^, and Phe^504^ (Kasahara et al., 1997; Kasahara and Kasahara, 2000) but a full protein alignment containing all the sequences analysed in this study revealed that these positions are not exclusively present in galactose transporters (Supplementary Figure S5). This observation indicates that the relevance of these sites cannot be universally extrapolated to the *K. marxianus* sequences. The phylogeny of the proteins (Figure 2) illustrated that there are distinct subclades that include proteins both positive and negative for galactose transport so alignments of proteins within each subclade were performed to see whether distinguishing amino acids could be identified. Comparison of the Lac12 sequences led to the identification of 38 amino acids that differ between the sequences of Lac12, Lac12-2 and Lac12-4, positive for galactose transport and Lac12-3, negative for galactose transport (Supplementary Figure S6). A similar analysis to the Kht sequences revealed 24 amino acids that could be critical for galactose transport in these proteins (Supplementary Figure S7). The most striking finding was that there is a unique position in these proteins that differs in a consistent way between KMXK_A02960, KMXK_A02940, KMXK_A02920 and Hgt1_*Kl*, which allow growth on 2% galactose, and KMXK_A02930, KMXK_A02950 and KMXK_E00380, which do not (Figure 6). In galactose transporting proteins, the residue at position 435 is alanine, whereas it is threonine in the proteins that do not transport this sugar. To test whether this residue plays a critical role in sugar recognition and transport, the threonine residue in KMXK_A02930 was replaced by alanine via site-directed mutagenesis. However, heterologous expression of this variant did not confer growth on galactose, indicating that this modification alone is not critical for galactose transport (data not shown).

## Discussion

### Expansion of Major Facilitator Superfamily Transporters in *K. marxianus*

In this study, it was shown that the phenomenon of duplicated proteins of the MFS superfamily in *K. marxianus* is not restricted to *LAC12*, but also includes the *KHT* and *HGT* gene families. In contrast to *LAC12*, where the duplications comprise individual genes distributed in different sub-telomeric regions, and with the exception of the *HGT*-like gene (KMXK_E00380), the additional *KHT* and *HGT* genes have arisen as tandem duplications. Within the *Kluyveromyces* genus, there are two copies of *KHT*, termed *KHT1* and *KHT2* in all species (data not shown), but the larger tandem duplications only occurred in *K. marxianus*, suggesting that some particular evolutionary pressure has driven this selection. It is intriguing to see that there is variability in the number of expanded genes among the seven sequenced strains we examined, demonstrating a certain amount of genome fluidity. Although the *K. marxianus* strains included in this study come from diverse environments and collections, all have been propagated for some time in laboratories or used for industrial processes. It is possible, therefore, that the copy number reductions, which have arisen by inter-gene homologous recombination, are a consequence of propagation in “non-natural” settings. In this regard, the phenomenon mirrors the well-known *KHT1* – *KHT2* recombination in *K. lactis* that gave rise to *RAG1* (Weirich et al., 1997). Indeed, many studies of sugar transport in *K. lactis* only consider *RAG1* (which mainly resembles *KHT2*) in the context of a low-affinity glucose transporter (Breunig et al., 2000). The alternative scenario, that laboratory selection led to the expansions, is not compatible with the same expansions and divergence being observed in completely independent strain lineages.

Gene duplication followed by functional divergence is a common evolutionary mechanism. The restriction to *K. marxianus* and the relatively high degree of sequence similarity point to these expansions being relatively recent events. Other studies of bacterial and eukaryotic genomes found that recent gene duplications usually encode proteins that are secreted or involved in membrane functions (Kondrashov et al., 2002), which is also the case for these *K. marxianus* MFS proteins. In additional to duplication, which allows proteins to acquire alternative functions, tandem arrays of genes of similar sequence allow intra-locus recombination, which can give rise to recombinants that may have novel functions or to loss of genes that are functionally redundant (Mendonça et al., 2011). Such recombinations were identified in some *K. marxianus* strains but no novel functions were identified for the recombinant proteins. Since, however, the phenotypic space assessed was restricted to glucose and galactose transport, other functions remain a possibility.

There is a dearth of knowledge on the natural ecology of *K. marxianus.* It has traditionally been considered a dairy yeast but whole genome comparisons and studies of Lac12p variation showed that only a subset of *K. marxianus* strains are adapted to this niche (Ortiz-Merino et al., 2018). Examination of the origins of *K. marxianus* strains in culture collections shows that the yeast can be isolated from plants, fruits and other non-dairy niches plant sources (Suzuki et al., 2014; Zhong et al., 2016). It can be speculated that the expansion and functional diversification of MFS sugar transporters has its origins in pressures in the evolutionary history of *K. marxianus.* Glucose limitation was shown to trigger duplication of the hexose transporters in *S. cerevisiae* (Brown et al., 1998). In that study, three tandem copies of a hybrid transporter formed by recombination between the *HXT6* and *HXT7* genes were found in an evolved strain. Further experiments showed that this strain outcompetes the parental strain in low glucose competition experiments. It is also known that cellular stress can be a driver of gene duplication and evolution of new functions (Kondrashov, 2012). It could be speculated that duplication of MFS genes in *K. marxianus* arose in a period of stress or nutrient limitation, with new functions being acquired later. It remains to be determined whether these duplications play any role in the particular traits of *K. marxianus*, such as thermotolerance and rapid growth (Lane and Morrissey, 2010).

### Functional Redundancy of Galactose Transporters in *K. marxianus*

The genetics of galactose transport has been well-studied in *K. lactis*, but it is now clear that the situation in *K. marxianus* is more complicated. There are also some common features. For example, in *K. lactis, HGT1* encodes a protein capable of transporting both glucose and galactose (Billard et al., 1996; Baruffini et al., 2006). The syntenic arrangement, high degree of sequence similarity, and functional analysis of KMXK_A02960 indicates that these genes are orthologous. It is interesting to note that a previous screen for xylose transporters from *K. marxianus* reported that KMXK_A02960 transports xylose and arabinose and named the gene *kmAXT1* (Knoshaug et al., 2015). That study did not report the duplicated genes and paralogs were not tested. Thus, it remains to be determined whether other Hgt1-like proteins can also transport pentose sugars. Regarding the hexose galactose, however, it was shown that two of the *HGT1*-like genes encode proteins capable of low-affinity transport, and one of high-affinity transport, and only the Hgt1 ortholog KMXK_A02960 has retained the capacity for glucose transport (Figure 4). It was also known from work on *K. lactis* that Lac12 is a low affinity galactose transporter, so it was not a surprise to find the same result in this study. The duplicated genes *LAC12-2* and *LAC12-4* also encode proteins that can transport galactose, but the unexpected finding was that Lac12-4 is also capable of supporting excellent growth on 0.1% galactose and is a high affinity galactose transporter (Figure 1). The *KHT* genes in *K. lactis* are thought to encode low affinity glucose transporters (Milkowski et al., 2001), though it must be considered that many of the studies were performed with a strain carrying *RAG1*, the recombined gene that contains the *KHT1* promoter and the 5′ sequence of *KHT1* along with most of *KHT2*. The possible role of *K. lactis* Kht1 and Kht2 in transporting galactose or other sugars does not seem to have received a lot of attention. In *K. marxianus*, there are three copies each of *KHT1* (two in strain CBS6556) and *KHT2*, all of which encode high affinity glucose transporters. Only one of the six Kht-like proteins, KMXK_E03670, also has the capacity to transport galactose. In total, eight *K. marxianus* proteins with some capacity to transport galactose when heterologously expressed in *S. cerevisiae* were identified, indicating a remarkable functional overlap. Despite this, the proteins are not completely redundant as shown by the analysis of strains carrying different combinations of inactivated genes. Inactivation of any one of the transporters (or set of similar transporters) did not have a discernible effect but any pairwise combination did affect growth on 0.1% galactose (Figure 5). In contrast, it was necessary to inactivate all identified galactose transporters to block growth on 2% galactose, consistent with the data that low affinity transporters were more prevalent than high affinity ones. Inferences as to whether particular transporters are high or low affinity are made based on whether they support growth on 0.1% galactose but detailed kinetic studies would be required to be definitive on this point. In general, high-affinity transporters are thought to be induced and allow growth when the substrate concentration is low, whereas low-affinity transporters, which require high substrate concentrations, can allow a higher uptake rate and flux. A study examining the kinetics of sugar transport in *K. marxianus* CCT7735 (UFV3) found two galactose transport systems; a low-affinity system present in lactose-grown cells and a second system able to transport glucose and galactose (da Silveira et al., 2018). It is possible that these two systems are comprised of several of the transporters identified in this study. For example, the *LAC12* genes could encode the low-affinity galactose transport system. This idea is supported by heterologous expression data (Figure 1) and data indicating that expression of these genes is induced by lactose and repressed by glucose (Varela et al., 2017b). The Hgt and the KMXK_E03670 Kht transporter could be part of the second transport system mentioned. However, studying the expression of these genes and the kinetics of galactose transport in *K. marxianus* mutants is required to understand the physiological role of these transporters. The range of substrates assessed could also be investigated since no substrates have yet been identified for some transporters, and it is also possible some of those shown here to transport glucose or galactose might actually have higher activity toward another substrate.

It is notoriously difficult to predict substrates for MFS sugar transporters and this family includes proteins able to transport various ions, sugar alcohols, and other molecules across the cell membrane (Lewinson et al., 2006). Having a set of proteins, some of which transport galactose and some of which do not offered a good opportunity to try to identify particular amino acids that might be important for distinguishing galactose from other sugars. Similar work has already been performed with *S. cerevisiae* Gal2, which diverged from an ancestral glucose permease. In that case, three critical amino acids were identified (Kasahara et al., 1997; Kasahara and Kasahara, 2000). From examination of the *K. marxianus* galactose transporters, however, it was immediately apparent that these residues are not diagnostic for galactose transporters in general (Supplementary Figure S5). In fact, there are no conserved amino acids that can be seen in all *K. marxianus* galactose transporters. Examination of the amino acid sequences within each sub-clade (Lac, Hgt, Kht) gave some clues as to what changes are required to enable galactose transport. This is most evident within the Hgt sub-clade where a single residue differentiates galactose and non-galactose transporters (Figure 6). However, the substitution of this residue in KMXK_A02930 (threonine to alanine substitution) was not sufficient to render it a galactose transporter (data not shown). This result is not completely unexpected considering that this position is a threonine in the galactose transporter KMXK_E03670 and is not a conserved alanine in other proteins that transport galactose (Supplementary Figure S5). Also, this residue is located in the 12th transmembrane domain, away from the pore that interacts with the substrate. Structural studies in the human transporter GLUT1, a MFS transporter that shares the topology and substrate recognition mechanism of yeast transporters, showed that the transmembrane segments 1, 2, 4, 5, 7, 8, 10, and 11 form the tunnel required for substrate transport (Kasahara et al., 2009; Sheena et al., 2011). Altogether, these findings suggest that sugar specificity in these proteins results from the combined contributions of multiple amino acids. It is apparent that different solutions can be found to create the correct environment in the substrate channel for galactose recognition and transport. Given the very close overall sequence similarity between these proteins, they will be a valuable tool for structural studies to model and identify exactly how galactose is selected for transport. Further investigations are needed to try to understand why *K. marxianus* has this array of transporters, for galactose and for other sugars.

## Author Contributions

JV, MP, and NM performed the experimental work. JV and JM wrote the manuscript and conceived the project.

## Conflict of Interest Statement

The authors declare that the research was conducted in the absence of any commercial or financial relationships that could be construed as a potential conflict of interest.

## Footnotes

1 http://www.cbs.dtu.dk/services/TMHMM/

## Supplementary Material

FIGURE S1 Map of the CRISPR-Cas9 plasmid used in this study. The pUCC001 plasmid was obtained by introducing a double-restriction site for the type IIS restriction enzyme, BsaI (gray shading) into the pUDP002 plasmid (Juergens et al., 2018). The desired target sequence can be chemically synthesized as a pair of complementary DNA oligos containing specific overhangs compatible with pUCC001. This oligo duplex can then be cloned between the HH ribozyme and the gRNA scaffold sequences (purple and blue shading, respectively) by Golden Gate assembly.

FIGURE S2 Recombination event leading to the formation of KMXK_E03660. **(A)** A scheme depicting the recombination event between the *KHT* genes, KMAR_50344 and KMAR_50343, is shown. The 5′ end of KMAR_50344 (gray) and the 3′ end of KMAR_50343 (purple) recombined to form KMXK_E03660 (red). **(B)** A DNA alignment of the KMAR_50344, KMAR_50343, and KMXK_E03660 sequences is shown. Three sections of the alignment are shown; the 5′ end of the three sequences, where KMXK_E03660 is identical to KMAR_50344 (top), the recombination point (middle) and the 5′ section of the gene where KMXK_E03660 is identical to KMAR_50343 (bottom). The different sequences are shown in colors as in the panel above. The exact recombination point is indicated with a red vertical line in position 371. Mismatches between the KMAR_50344/KMAR_50343 sequences and KMXK_E03660 are shown in bold.

FIGURE S3 Recombination event leading to the formation of KLMA_10546. **(A)** A scheme depicting the recombination event between KMXK_A02950 and KMXK_A02930 is shown. The 5′ end of KMXK_A02950 (gray) and the 3′ end of KMXK_A02930 (purple) recombined to form KLMA_10546 (red). **(B)** A DNA alignment of these sequences is shown as in Supplementary Figure S2. The exact recombination point is indicated with a red vertical line in position 230. Mismatches between the KMXK_A02950/KMXK_A02930 sequences and KLMA_10546 are shown in bold.

FIGURE S4 Construction of an *HGT* null mutant in *K. marxianus* NBRC1777. The CRISPR-*Cas9* system was used to induce mutagenesis of the *HGT* genes by targeting the 5′ CGCTCCAACCTGGGGTAT 3′ sequence present in all *HGT* genes with a single gRNA. **(A)** Scheme showing the repair event occurred upon targeting the *HGT* genes with the CRISPR-*Cas9* system. Recombination between the two ends of the *HGT* loci is shown by gray shading. Stop codons in the recombinant *HGT*, obtained as consequence of the deletion, are represented by a red line. Primers used to screen the mutant colonies are shown as red arrows. **(B)** Screening of *hgt* mutant colonies. Transformants were screen by PCR using the Diag*HGT* primers. Lane 1, NBRC1777 wild-type. Lanes 2 and 3, NBRC1777 strains transformed with the pUCC001-*HGT* plasmid. DNA ladder Hyperladder I. **(C)** Recombination events in mutants *hgt75* (top panel) and *hgt77* (bottom panel). DNA sequence alignments between the wild-type and mutant sequences are shown. Blue and purple boxes represent the genes A02960 and A02920 where the recombination events took place. Coordinates in the NBRC1777 genome (chromosome I) are shown as numbers above the alignments. The protospacer sequence is shown in bold.

FIGURE S5 Multiple sequence alignment of Lac12, Kht, Hgt, and Gal2 sequences. The protein sequence alignment was generated using MUSCLE 3.8 and visualised in Jalview 2. The *S. cerevisiae* Gal2 transporter was included in this analysis as the three amino acid positions critical for galactose transport have been identified in this transporter. These residues (Tyr^352^, Tyr^446^, and Phe^504^) are marked in red in the alignment.

FIGURE S6 Multiple sequence alignment of Lac12 sequences. The multiple sequence alignment was generated using MUSCLE 3.8 and imported into Jalview 2 for visualisation. Sequence differences between galactose transporters (Lac12_Km, Lac12-2_Km, and Lac12-4_Km) and Lac12-3, unable to transport galactose, are marked in purple.

FIGURE S7 Multiple sequence alignment of *Kht* sequences. The sequence alignment was computed as described in Supplementary Figures S6, S7. Sequence differences between the galactose transporter KMXK_E03670 and the rest of the proteins are shown in purple.

TABLE S1 Annotation of putative MFS transporters in *K. marxianus* and *K. lactis.*

TABLE S2 Equivalence of KHT and HGT genes in different K. marxianus genomes. Genes marked with an asterisk have arisen by recombination between two genes.
