# Supplementary figures and images for: Expansion and Diversification of MFS Transporters in Kluyveromyces marxianus
Source: Front Microbiol. 2019 Jan 10;9:3330. doi: 10.3389/fmicb.2018.03330 (PMC6335341; doi:10.3389/fmicb.2018.03330)

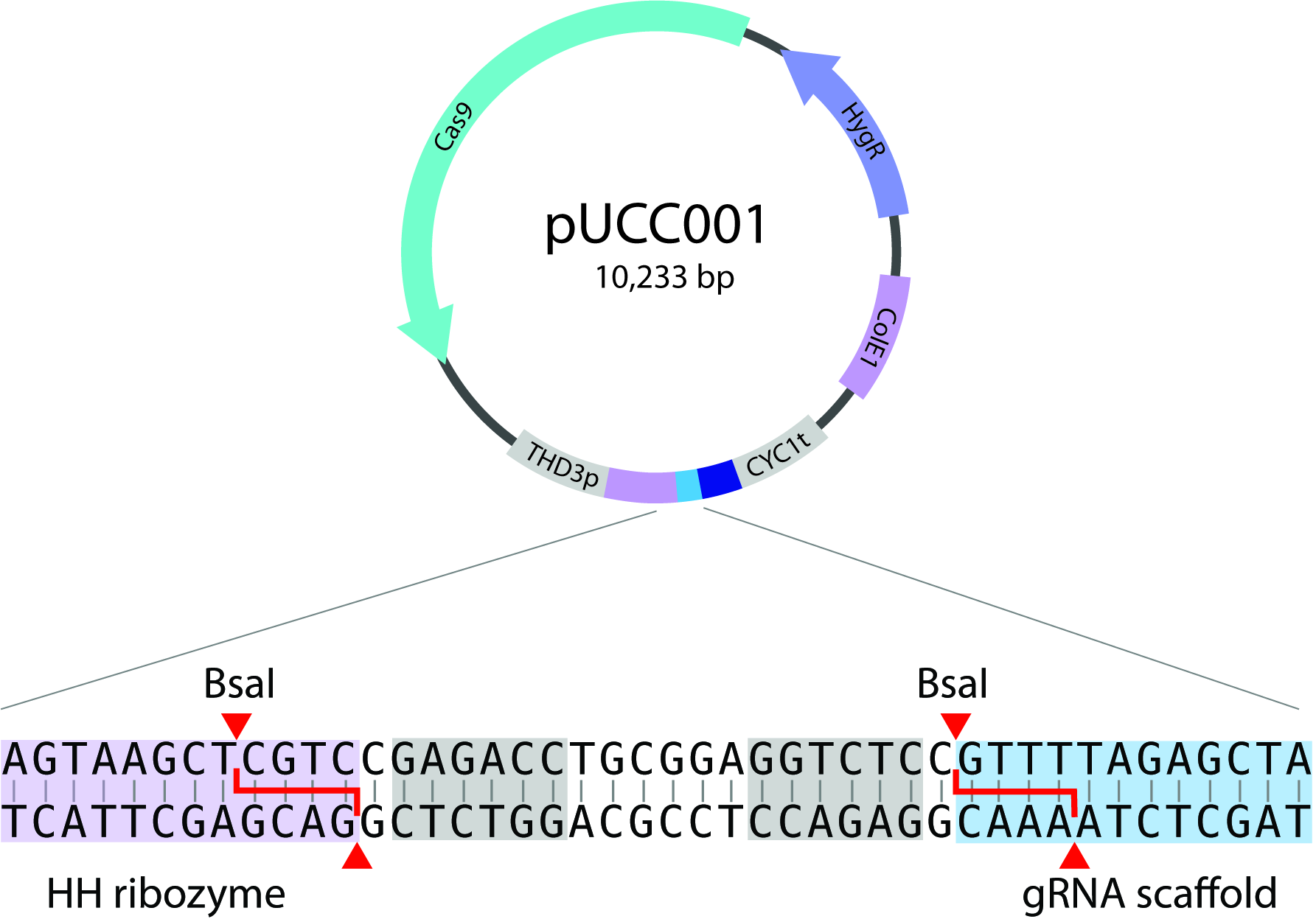

Supplement: FIGURE S1 — Map of the CRISPR-Cas9 plasmid used in this study. The pUCC001 plasmid was obtained by introducing a double-restriction site for the type IIS restriction enzyme, BsaI (gray shading) into the pUDP002 plasmid (Juergens et al., 2018). The desired target sequence can be chemically synthesized as a pair of complementary DNA oligos containing specific overhangs compatible with pUCC001. This oligo duplex can then be cloned between the HH ribozyme and the gRNA scaffold sequences (purple and blue shading, respectively) by Golden Gate assembly. [file Image_1.TIF]

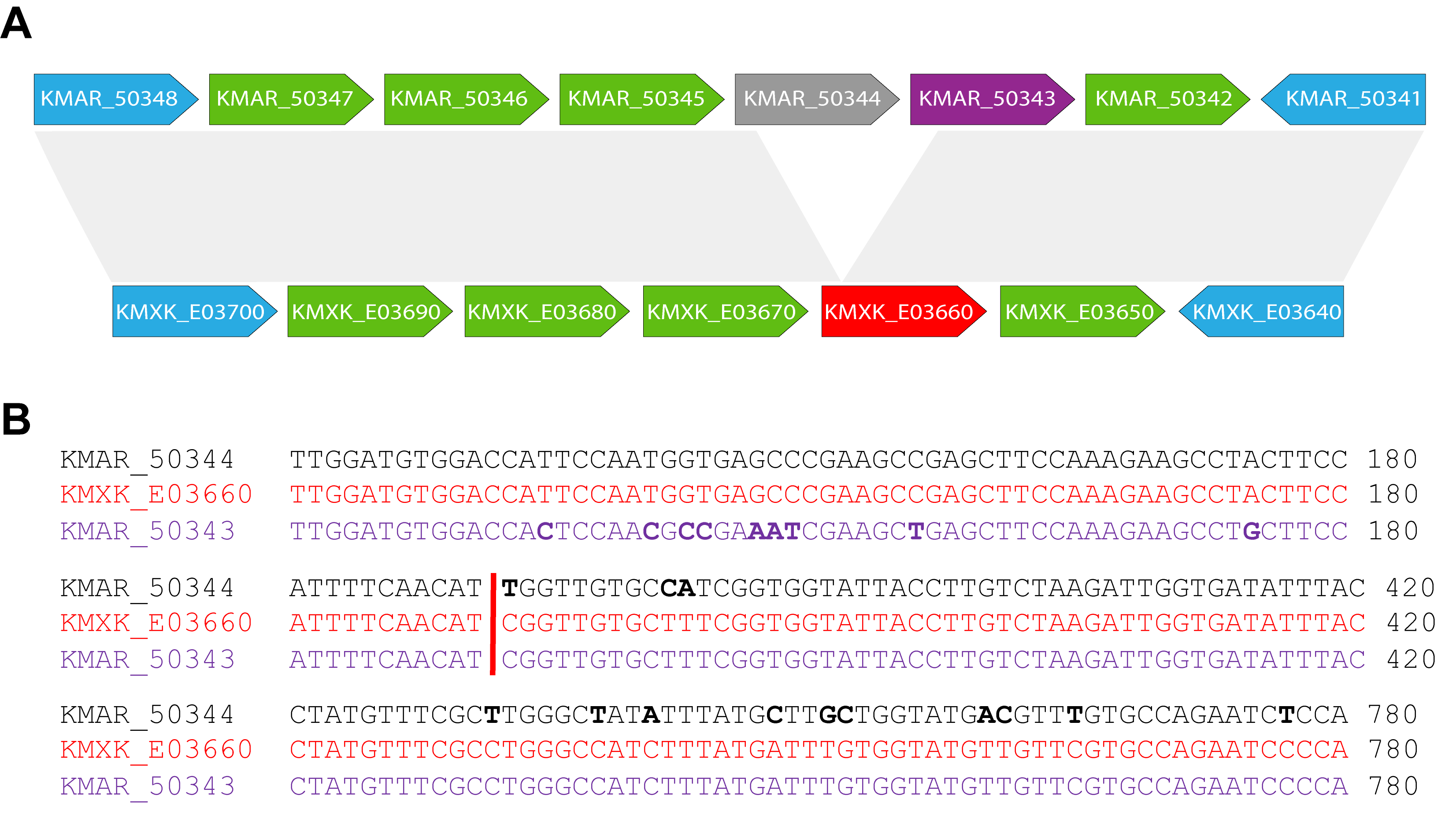

Supplement: FIGURE S2 — Recombination event leading to the formation of KMXK_E03660. (A) A scheme depicting the recombination event between the KHT genes, KMAR_50344 and KMAR_50343, is shown. The 5′ end of KMAR_50344 (gray) and the 3′ end of KMAR_50343 (purple) recombined to form KMXK_E03660 (red). (B) A DNA alignment of the KMAR_50344, KMAR_50343, and KMXK_E03660 sequences is shown. Three sections of the alignment are shown; the 5′ end of the three sequences, where KMXK_E03660 is identical to KMAR_50344 (top), the recombination point (middle) and the 5′ section of the gene where KMXK_E03660 is identical to KMAR_50343 (bottom). The different sequences are shown in colors as in the panel above. The exact recombination point is indicated with a red vertical line in position 371. Mismatches between the KMAR_50344/KMAR_50343 sequences and KMXK_E03660 are shown in bold. [file Image_2.TIF]

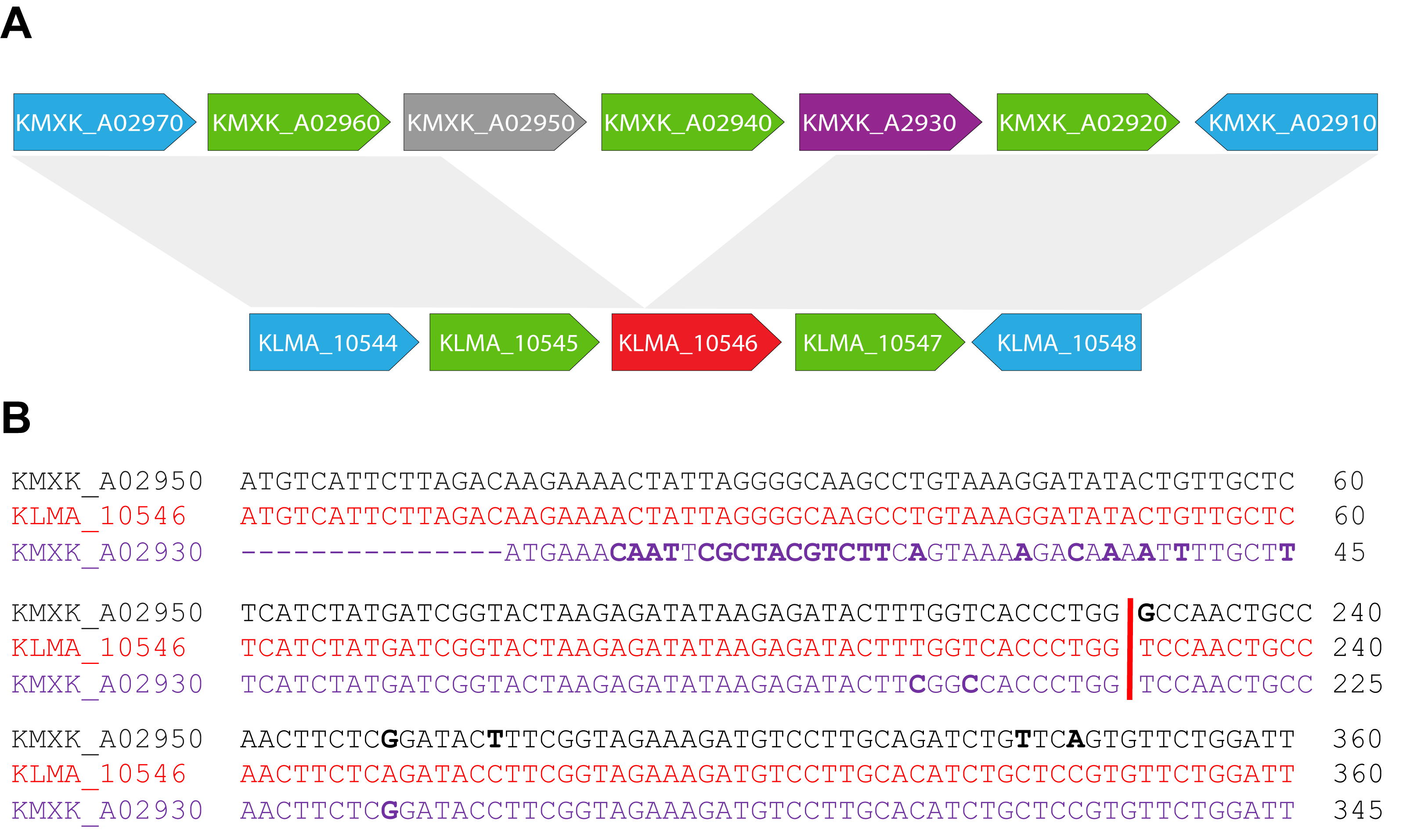

Supplement: FIGURE S3 — Recombination event leading to the formation of KLMA_10546. (A) A scheme depicting the recombination event between KMXK_A02950 and KMXK_A02930 is shown. The 5′ end of KMXK_A02950 (gray) and the 3′ end of KMXK_A02930 (purple) recombined to form KLMA_10546 (red). (B) A DNA alignment of these sequences is shown as in Supplementary Figure S2. The exact recombination point is indicated with a red vertical line in position 230. Mismatches between the KMXK_A02950/KMXK_A02930 sequences and KLMA_10546 are shown in bold. [file Image_3.TIF]

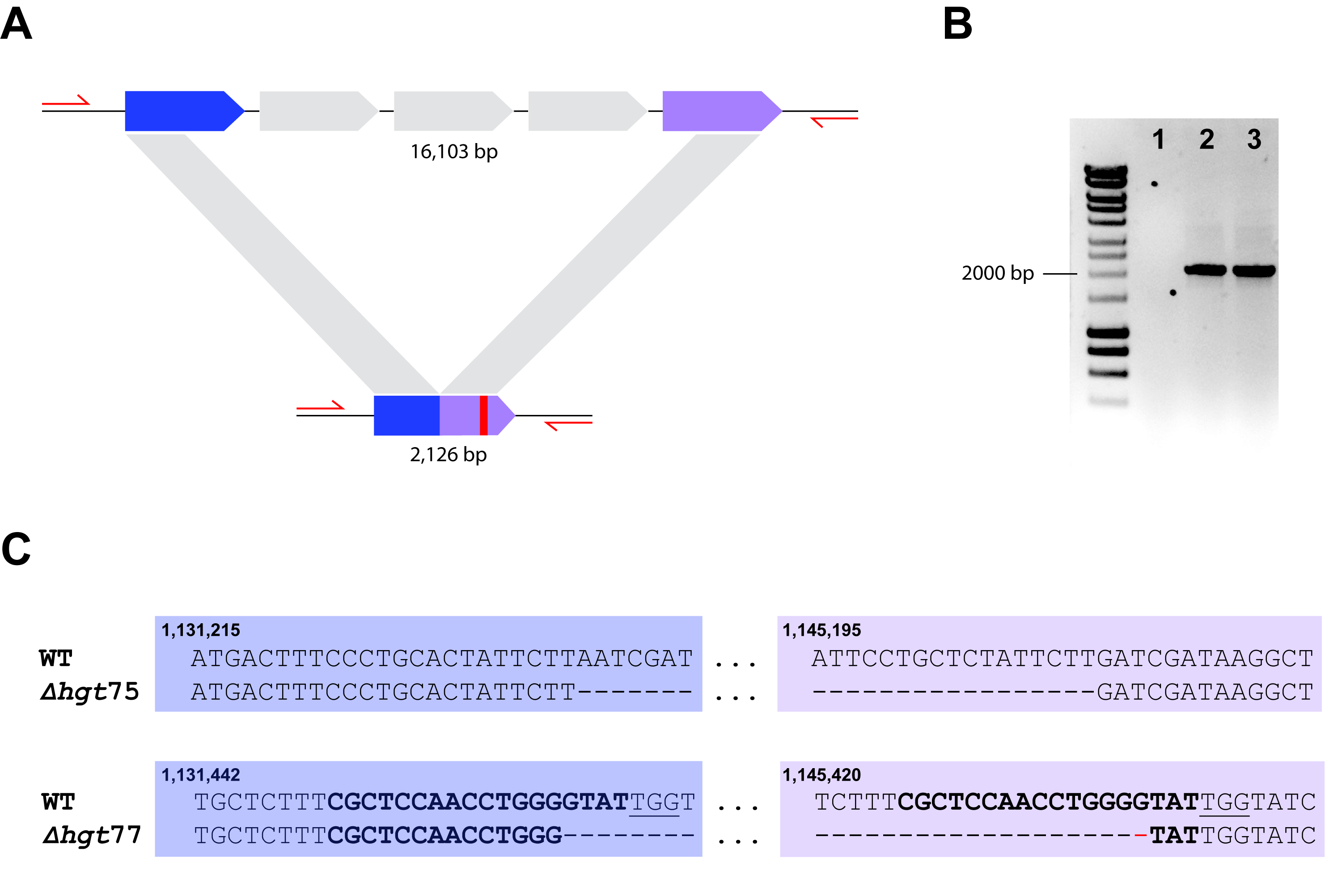

Supplement: FIGURE S4 — Construction of an HGT null mutant in K. marxianus NBRC1777. The CRISPR-Cas9 system was used to induce mutagenesis of the HGT genes by targeting the 5′ CGCTCCAACCTGGGGTAT 3′ sequence present in all HGT genes with a single gRNA. (A) Scheme showing the repair event occurred upon targeting the HGT genes with the CRISPR-Cas9 system. Recombination between the two ends of the HGT loci is shown by gray shading. Stop codons in the recombinant HGT, obtained as consequence of the deletion, are represented by a red line. Primers used to screen the mutant colonies are shown as red arrows. (B) Screening of hgt mutant colonies. Transformants were screen by PCR using the DiagHGT primers. Lane 1, NBRC1777 wild-type. Lanes 2 and 3, NBRC1777 strains transformed with the pUCC001-HGT plasmid. DNA ladder Hyperladder I. (C) Recombination events in mutants hgt75 (top panel) and hgt77 (bottom panel). DNA sequence alignments between the wild-type and mutant sequences are shown. Blue and purple boxes represent the genes A02960 and A02920 where the recombination events took place. Coordinates in the NBRC1777 genome (chromosome I) are shown as numbers above the alignments. The protospacer sequence is shown in bold. [file Image_4.TIF]

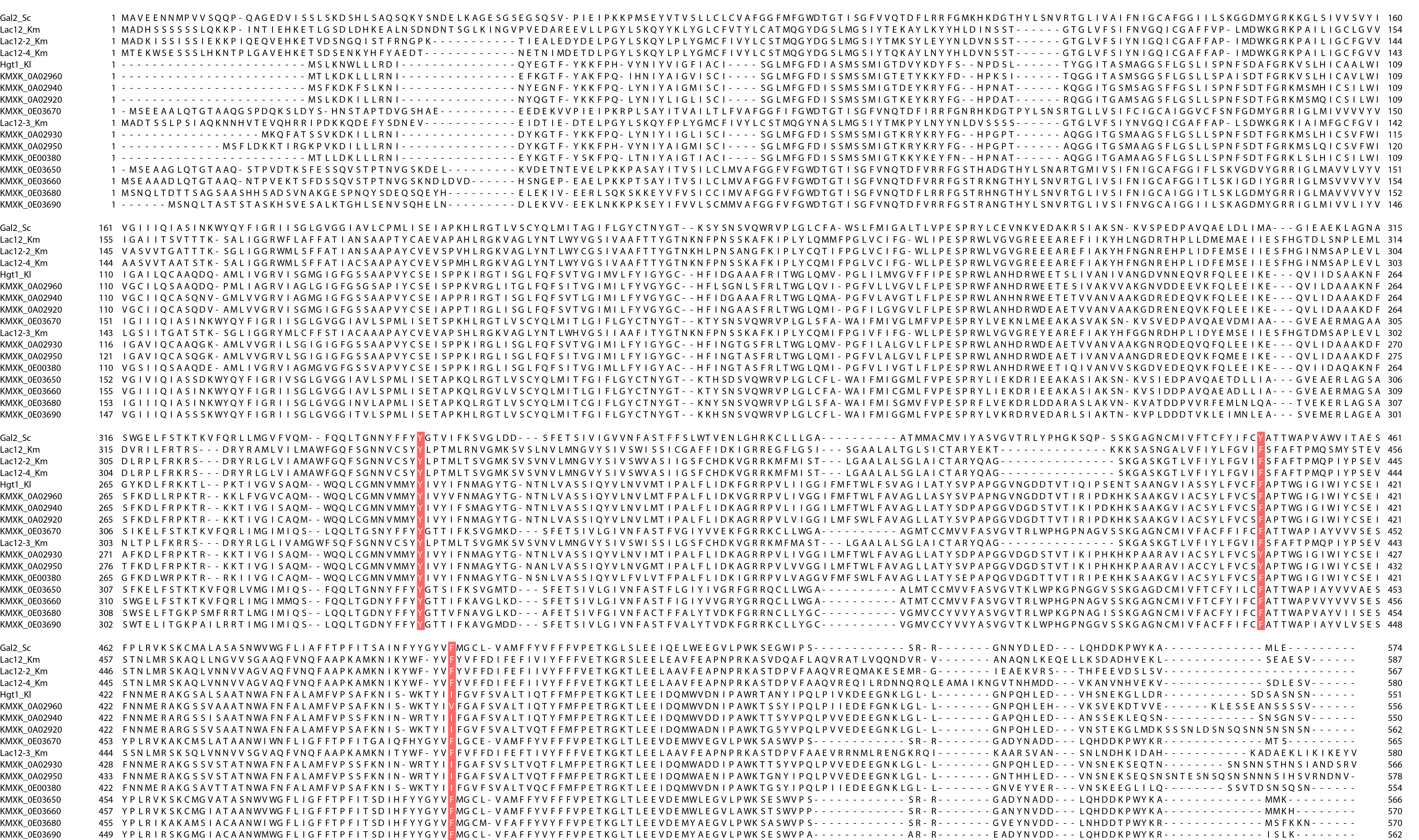

Supplement: FIGURE S5 — Multiple sequence alignment of Lac12, Kht, Hgt, and Gal2 sequences. The protein sequence alignment was generated using MUSCLE 3.8 and visualised in Jalview 2. The S. cerevisiae Gal2 transporter was included in this analysis as the three amino acid positions critical for galactose transport have been identified in this transporter. These residues (Tyr352, Tyr446, and Phe504) are marked in red in the alignment. [file Image_5.JPEG]

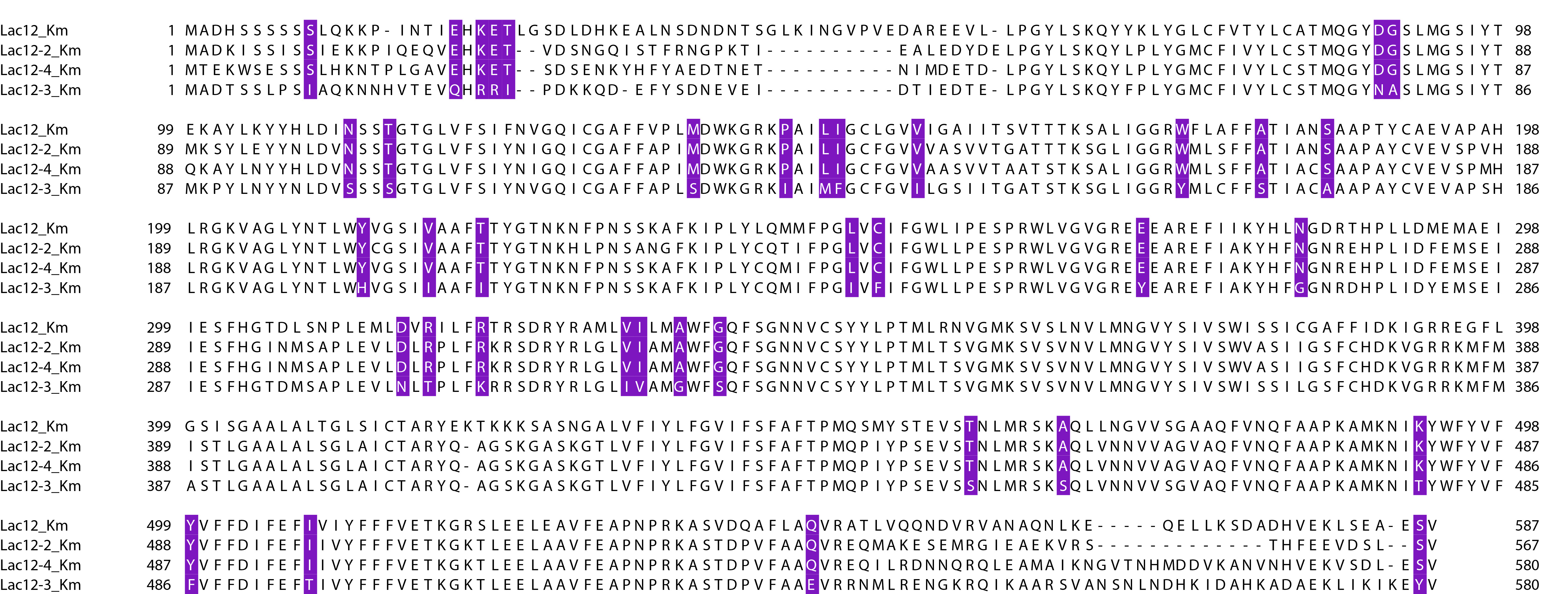

Supplement: FIGURE S6 — Multiple sequence alignment of Lac12 sequences. The multiple sequence alignment was generated using MUSCLE 3.8 and imported into Jalview 2 for visualisation. Sequence differences between galactose transporters (Lac12_Km, Lac12-2_Km, and Lac12-4_Km) and Lac12-3, unable to transport galactose, are marked in purple. [file Image_6.JPEG]

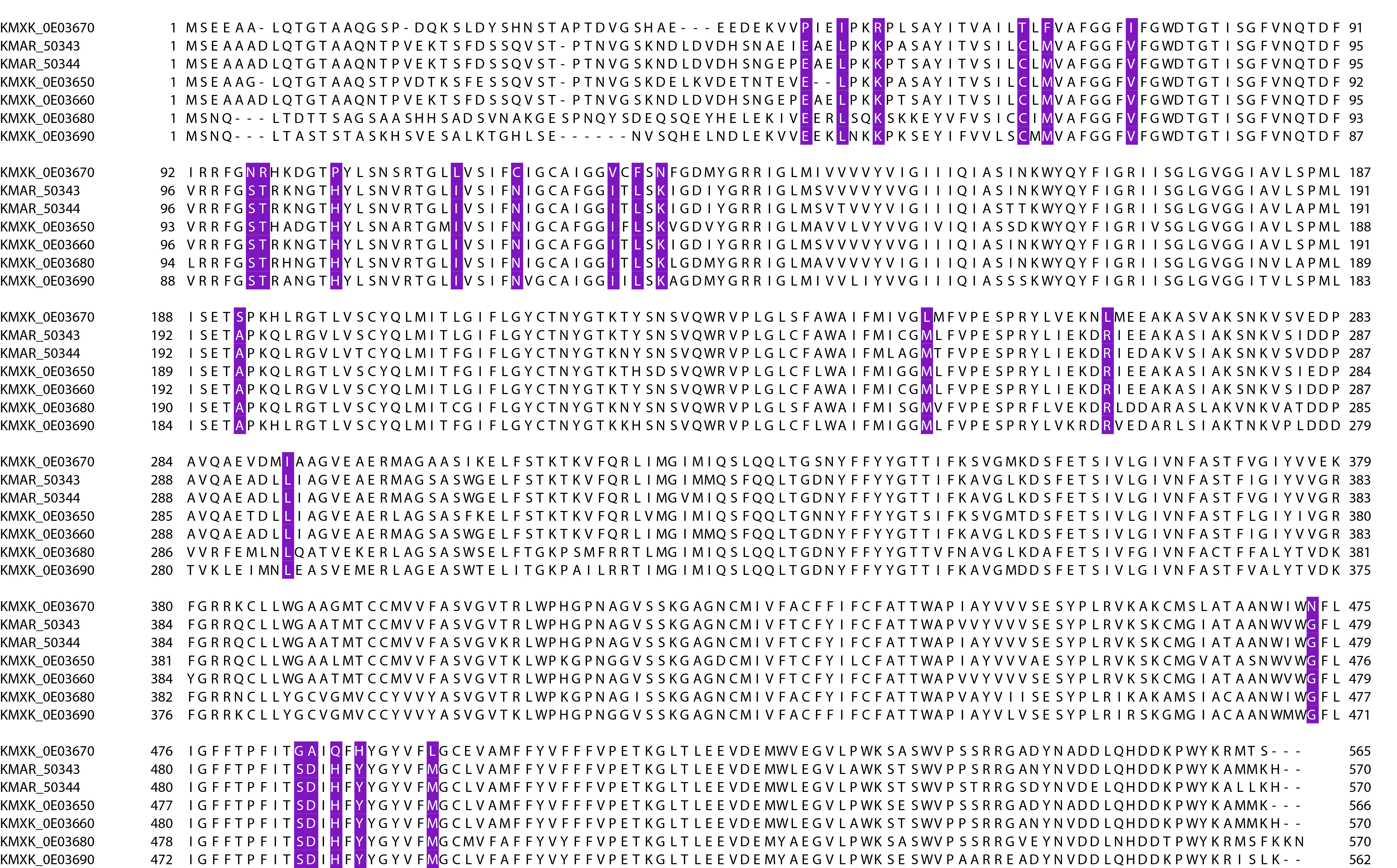

Supplement: FIGURE S7 — Multiple sequence alignment of Kht sequences. The sequence alignment was computed as described in Supplementary Figures S6, S7. Sequence differences between the galactose transporter KMXK_E03670 and the rest of the proteins are shown in purple. [file Image_7.JPEG]
