# Supplementary material for: Expansion and Diversification of MFS Transporters in Kluyveromyces marxianus
Source: Front Microbiol. 2019 Jan 10;9:3330. doi: 10.3389/fmicb.2018.03330 (PMC6335341; doi:10.3389/fmicb.2018.03330)
Supplement: TABLE S1 — Annotation of putative MFS transporters in K. marxianus and K. lactis. [file Table_1.DOCX]

**Table S1.** Annotation of putative MFS transporters in *K. marxianus* and *K. lactis*

|  | *K. marxianus* CBS6556 | *K. lactis* CBS2359 |
| --- | --- | --- |
| Total number of proteins | 5,117 | 5,076 |
| Hit with TransportDB | 510 | 502 |
| Containing >3 TM domians | 269 | 270 |
| Predicted MFS transporters | 49 | 41 |

**Table S2.** Equivalence of *KHT* and *HGT* genes in different *K. marxianus* genomes. Genes marked with an asterisk have arisen by recombination between two genes.

|  | CBS 6556 | NBRC 1777 | DMKU3-1042 |
| --- | --- | --- | --- |
| *HGT*-like | KMXK_A02920 | KMAR_10531 | KLMA_10547 |
| *HGT*-like | KMXK_A02930 | KMAR_10530 | KLMA_10546* |
| *HGT*-like | KMXK_A02940 | KMAR_10529 | - |
| *HGT*-like | KMXK_A02950 | KMAR_10528 | KLMA_10546* |
| *HGT*-like | KMXK_A02960 | KMAR_10527 | KLMA_10545 |
| *KHT*-like | KMXK_E03650 | KMAR_50342 | KLMA_50360 |
| *KHT*-like | KMXK_E03660* | KMAR_50343 | KLMA_50361 |
| *KHT*-like | KMXK_E03660* | KMAR_50344 | KLMA_50361 |
| *KHT*-like | KMXK_E03670 | KMAR_50345 | KLMA_50362 |
| *KHT*-like | KMXK_E03680 | KMAR_50346 | KLMA_50363 |
| *KHT*-like | KMXK_E03690 | KMAR_50347 | KLMA_50364 |
| *KHT*-like | KMXK_E00380 | KMAR_50027 | KLMA_50032 |
